# Association of Clinical Severity in Autism Spectrum Disorder with Biomolecules Involved in Lipid Metabolism, Inflammation and miRNAs

**DOI:** 10.3390/biom16020303

**Published:** 2026-02-14

**Authors:** Maria Gevezova, Michael Maes, Iliana Pacheva, Nikolay Mehterov, Zdravko Ivanov, Elena Timova, Stefka Spassieva, Erhard Bieberich, Maria Kazakova, Ivan Ivanov, Victoria Sarafian

**Affiliations:** 1 Department of Medical Biology, Medical University of Plovdiv, 4002 Plovdiv, Bulgaria; 2 Research Institute, Medical University of Plovdiv, 4002 Plovdiv, Bulgaria; 3 Sichuan Provincial Center for Mental Health, Sichuan Provincial People’s Hospital, School of Medicine, University of Electronic Science and Technology of China, Chengdu 610072, China; 4 Department of Paediatrics, Medical University of Plovdiv, 4002 Plovdiv, Bulgaria; 5 Paediatrics Clinic, St. George University Hospital, 4002 Plovdiv, Bulgaria; 6 Department of Physiology, University of Kentucky, Lexington, KY 40536, USA

**Keywords:** ASD, sphingomyelin phosphodiesterases, ceramide synthases, LAMP 1/2, miR-143-3p, miR-181a-5p

## Abstract

Autism spectrum disorder (ASD) is a heterogeneous neurological condition with an unclear etiology and pathogenesis. In recent years, studies have identified changes in lipid metabolism, inflammation, mitochondrial dysfunction, and mitophagy in patients with ASD. However, the specific interactions between these molecular signatures and their clinical applications in ASD remain largely unexplored. The aim of our study is to search for correlations between changes in gene and miRNA expression and the clinical characteristics of ASD. The investigation included a cohort of children with idiopathic ASD and healthy controls (HC). Diagnosis was established based on ADOS assessment (autism diagnostic observation schedule). Gene expression levels of sphingomyelin phosphodiesterases (SMPD1 and 5), ceramide synthases (CerS1 and 6), cyclooxygenase-2 (COX2), chitinase-3-like protein 1 (YKL40), and lysosome-associated membrane proteins 1 and 2 (LAMP1 and 2) were assessed using qPCR. The TaqMan assay was used for the quantification of miR-143-3p and miR-181a-5p. Our findings provide novel data on altered expression profiles of molecules related to lipid metabolism and LAMP1/2 in patients with ASD. We observed increased mRNA levels of CerS1, SMPD5, COX2, YKL40, LAMP1, and LAMP2 and decreased expression of miRNA-181a-5p in ASD patients compared to HC. Additionally, we identified a correlation between CerS1, CerS6, COX2, and miRNA-143-5p with ADOS scores. Multiple regression analysis revealed that 48.0% of the variance in the total ADOS score was explained by the combined effects of COX2, miRNA-143-3p, CerS1, CerS6 and age. These results provide new insights into the molecular alterations associated with ASD and may reinforce future studies aimed at clarifying their functional relevance.

## 1. Introduction

Autism spectrum disorder (ASD) encompasses a group of complex neurobehavioral and neurological conditions that are typically manifested in early childhood. Despite advances in understanding the etiology of ASD, its diagnosis remains reliant on the evaluation of behavioral symptoms, including reduced social interaction and repetitive, stereotyped behaviors [1]. Consequently, the identification of objective biological markers associated with ASD diagnosis and clinical severity is essential. This need is underscored by the rising prevalence of ASD, which ranges from 0.38% to 1.55% in Europe [2], with males being four times more affected than females [3].

While the multifactorial etiology of ASD remains largely elusive, numerous studies suggest that children with autism exhibit genetic variations, distinct metabolic profiles, and abnormalities in lipid metabolism [4,5,6,7]. These characteristics vary with age, gender, and symptom severity [8,9,10].

The metabolic phenotype of ASD is marked by decreased levels of anti-inflammatory and antioxidant molecules, coupled with an increase in stress-responsive metabolites such as ceramides, lactate, and glycerol [11,12].

Accumulating evidence indicates that ASD is not solely a neurodevelopmental disorder, but rather a systemic condition involving several interrelated biological domains: (1) inflammation and immune dysregulation; (2) altered lipid metabolism and ceramide synthesis; and (3) dysregulation of microRNAs (miRNAs) that modulate these processes.

(1) Inflammation and immune dysregulation

Inflammatory signaling is increasingly recognized as a central factor in the pathogenesis of autism, with the pathways involved in various neuroinflammatory and neurodegenerative conditions being mediated by lipid intermediates such as ceramide and sphingosine-1-phosphate. Furthermore, enzymes such as cyclooxygenase 2 (COX2), sphingosine kinases, and sphingomyelinase are involved in interconnected signaling networks that can lead to neuroinflammation and neurodegeneration [13,14,15].

YKL40, a glycoprotein member of “mammalian chitinase-like protein”, has been studied for its possible function in tissue remodeling. It was found that its expression increases in neuroinflammatory conditions [16,17]. The glycoprotein YKL40 may regulate neuroplasticity, regenerative processes in neurons and it had the potential to modulate neurotrophic factor-related changes in neuronal repair and regeneration [18]. Given its dual role in immune activation and tissue repair, YKL40 represents a potential bridge between inflammatory and neuroplastic mechanisms in ASD.

Other inflammation-related molecules include lysosome-associated membrane proteins 1 and 2 (LAMP1, LAMP2), which participate in autophagy and intracellular protein degradation. They are lysosomal-type transmembrane proteins whose function is related not only to the maintenance of the structural integrity of lysosomes and pH stability, but they are involved in immune dysregulation and gene regulatory networks including the mTOR–lysosome axis [19,20,21]. Dysregulation of this axis may enhance both inflammatory signaling and impaired autophagic flux, further linking immune dysfunction to cellular metabolism.

(2) Lipid metabolism and ceramide synthesis

**Figure 1:**
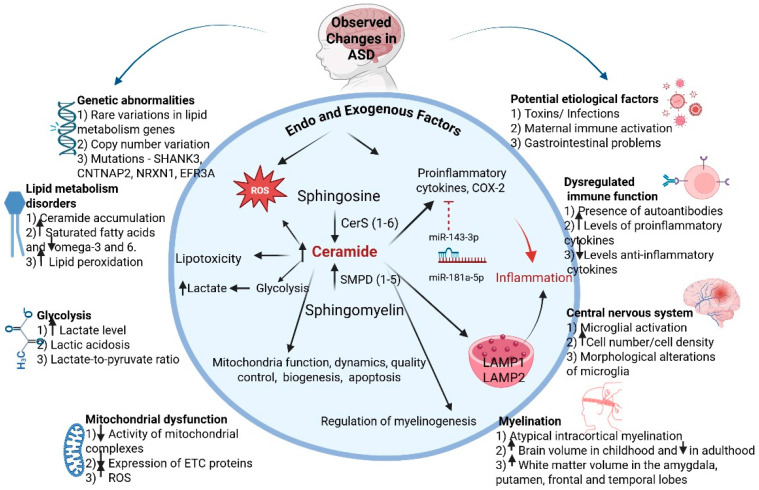
A generalized schematic representation of the mechanisms associated with ceramide metabolism and their connection to pathogenetic mechanisms in ASD (BioRender.com). Abbreviations: ROS—reactive oxygen species; ETC—electron transport chain; CerS—ceramide synthase; SMPD—sphingomyelin phosphodiesterase; COX2—cyclooxygenase-2; LAMP1/2—lysosome-associated membrane proteins 1 and 2.

Ceramides, which are central to sphingolipid metabolism, play a critical role in central nervous system (CNS) disorders. They have been shown to induce neuronal apoptosis through mitochondrial dysregulation and excessive production of reactive oxygen species (ROS) [14,22,23,24]. In mammals, ceramides are synthesized de novo by six ceramide synthases (CerS 1 to 6). Ceramides can also be generated from five sphingomyelin by sphingomyelin phosphodiesterases (SMPD1 to 5). They participate in numerous signaling pathways and can be modulated in response to cellular stress [25,26,27]. In addition, mutations in lysosomal acid sphingomyelinase (ASM; SMPD1) lead to accumulation of lipid substrates (sphingomyelin) in the lysosomes of cells (Niemann-Pick Disease type A and B) [28]. This is followed by dysfunction of different organs and a variety of clinical symptoms resulting from multiple cell damage in the affected cells [28]. Even more interesting is sphingomyelin phosphodiesterase 5 (SMPD5 or MA-nSMase) localized in mitochondria with a primary role in the hydrolysis of sphingomyelin to ceramide and phosphocholine [29,30]. Sphingomyelinases can be activated in response to oxidative stress, which leads to an increase in ceramide levels and induces mitochondrial disfunction [31]. Together, these mechanisms form a pathophysiological network in which lipid dysregulation contributes to neuroinflammation, oxidative stress, and neuronal damage, as summarized in Figure 1.

CerS (1 to 6) exhibit significant differences in their biological functions, as they synthesize ceramides with varying fatty acid chain length moieties and have different tissue and cell type distribution [32] (Table S1, Supplementary File). Such molecular diversity may explain region-specific metabolic vulnerability in ASD, linking altered lipid homeostasis to neuronal dysfunction and behavioral symptoms. Collectively, these findings suggest that inflammation, lipid metabolism, and mitochondrial stress are not independent events but interrelated processes contributing to ASD pathogenesis (Figure 1). Even more remarkably, several miRNAs regulating immune responses, lipid metabolism, and mitophagy also show impaired expression in ASD.

(3) miRNA regulation of inflammation, lipid metabolism, and lysosomal function

Despite advances in understanding ceramide formation pathways, little is known about the regulation by microRNAs (miRNAs) under different physiological or pathological conditions. miRNAs are small regulatory RNA molecules synthesized by cells and used as post-transcriptional regulators of gene expression [33]. Moreover, their stability in various biological samples makes them ideal biomarkers with diagnostic and therapeutic significance [34]. In recent years, studies have shown that many immune-related miRNAs, as well as those regulating genes involved in lipid metabolism and mitophagy, exhibit aberrant expression in ASD [35,36].

Here, we propose an integrative framework connecting inflammation, lipid metabolism, lysosomal activity, and miRNA regulation in ASD. Nevertheless, the specific interactions among the investigated molecular signatures and their clinical application in ASD are largely unexplored. Hence, we study the key enzymes involved in ceramide metabolism, their potential regulation by miRNAs, and their association with inflammation. Specifically, we analyzed the gene expression patterns of sphingomyelin phosphodiesterases (SMPD1 and SMPD5), ceramide synthases (CerS1 and CerS6), COX2 and LAMP1/2, Chitinase-3-like protein 1 (CHI3L1, also known as YKL-40), alongside miR-143-3p and miR-181a-5p, two microRNAs associated with inflammation and metabolism. Hypothesizing that dysregulated lipid homeostasis in children with ASD could influence the severity of clinical symptoms, we also examined correlations between the expression profiles of molecules related to inflammation, autophagy and lipid metabolism with clinical ASD indicators.

We hypothesize that alterations in lipid metabolism are associated with mitochondrial dysfunction and metabolic imbalance, contributing to oxidative stress-related mechanisms in ASD. In this context, reactive oxygen species (ROS) are chemically reactive molecules derived from oxygen and are closely linked to mitochondrial activity and lipid metabolic pathways.

The differential intracellular localization of accumulated lipid substrates in cells with high energy demands (immune, neural, and muscle cells) may further contribute to the biological heterogeneity observed in ASD. This hypothesis is supported by evidence that ketogenic dietary interventions and related supplements can significantly improve ASD symptoms [37,38]. In addition to dietary carbohydrate restriction, ketogenic approaches are often combined with supplements such as medium-chain triglycerides, omega-3 fatty acids, and carnitine, which support mitochondrial fatty acid oxidation, bioenergetic efficiency, and anti-inflammatory signaling [36]. Collectively, these interventions may reduce inflammatory signaling and oxidative stress while improving mitochondrial function.

Based on this rationale, we examined the gene expression signatures of the key molecules involved in these pathways and their associations with clinical parameters and ADOS scores in children with ASD.

## 2. Materials and Methods

### 2.1. Participants

The study included a cohort of Bulgarian children with idiopathic ASD and healthy controls (HC). The diagnosis of ASD was established based on the DSM-5 criteria (Diagnostic and Statistical Manual of Mental Disorders) [1] following a comprehensive historical, physical, and neurological evaluation by a team of pediatric neurologists and psychiatrists, psychologists, and a specialist certified in ADOS assessment. All children with ASD underwent the ADOS-2 test at the Pediatrics Clinic of the University Hospital “St. George” to evaluate communication, social interaction, and play skills.

Participants in both the ASD and the HC groups were included in the study based on the following criteria:

1. Age range of 1.9 to 11 years.
2. No intake of vitamins, mineral supplements, immunomodulators, antibiotics, or similar substances in the past three months.
3. Absence of acute illnesses or epileptic seizures.
4. No concurrent diseases, gastrointestinal disorders, or chronic conditions such as infections, bronchial asthma, or diabetes mellitus.

### 2.2. Pre-Selection of Biomarkers

This was not an untargeted or targeted lipidomic, metabolomic or transcriptomic study in which many metabolites or transcripts were measured. Rather, we pre-specified a priori a few biomarkers which (as explained in the introduction) may be associated with the inflammatory and metabolic pathways in ASD. Phrased differently, we selected biomarkers (n = 10) to be analyzed and assayed as a focused hypothesis-driven panel.

Two microRNAs were selected based on target gene analysis and a review of the literature, which identified multiple miRNAs with altered expression in ASD. miR-181a-5p was chosen due to its involvement in regulating the inflammatory response, T-cell differentiation, and mitochondrial function [39]. Additionally, the miR-181a-5p profile has been suggested as a biomarker for immune-mediated inflammation in ASD [35,39]. 3′-UTR (3′-Untranslated Region) analysis of SMPD1, SMPD5, CerS1, CerS6, COX2, YKL40 and LAMP1/2 using mirWalk (http://mirwalk.umm.uni-heidelberg.de/; accessed on 15 September 2024) [40] TargetScan (http://www.targetscan.org/vert_80/; accessed on 15 September 2024) [41], and miRmap (https://mirmap.ezlab.org/; accessed on 15 September 2024) [42] prediction tools revealed that miR-143-3p has a binding site in three of these genes (LAMP1, CerS6, and COX2) (binding probability > 0.8). Therefore, these two miRNAs were selected for transcriptional analysis based on the reported abnormalities in ASD (miR-181a-5p) and the possible regulatory role over LAMP1, CerS6, and COX2, miR-143-3p and miR-181a-5p. Thus, gene expression analysis was performed on a targeted set of 8 protein-coding genes: CERS1, CERS6, SMPD1, SMPD5, COX2, YKL40, LAMP1, and LAMP2, selected a priori based on their putative role in biological pathways in ASD, as explained above and in the introduction.

### 2.3. Isolation of Total RNA from White Blood Cells (WBCs)

Blood samples were collected from participants following written informed consent from their parents, in compliance with the guidelines of the University Ethics Committee (Protocol No. 02/07.02.2024). Samples were drawn into EDTA-containing Vacutainer monovettes (S-Monovette 2.6 mL, Z-Sarstedt, Sarstedt AG & Co. KG, Nümbrecht, Germany) via venipuncture.

After centrifugation at 3000 rpm for 10 min, plasma and blood cells were separated, and erythrocytes were lysed using an ammonium chloride-based buffer (3.4 mM NH_4_HCO_3_, 155 mM NH_4_Cl, 96.7 µM EDTA). Total RNA was isolated from white blood cells (WBCs) using Trizol reagent (Thermo Fisher Scientific, Waltham, MA, USA, Lot No. 1559602) according to the manufacturer’s instructions. Following RNA extraction, samples were treated with the DNA TURBO kit (Thermo Fisher Scientific, Waltham, MA, USA, Lot No. AM1907) to remove residual DNA.

The RNA concentration and purity were quantified at 260/280 nm using the NanoDrop Nucleic Acid Quantification system (Thermo Fisher Scientific, Waltham, MA, USA). All samples were stored at −80 °C until further analysis.

### 2.4. Reverse Transcription and qPCR

Two micrograms (μg) of total RNA were reverse transcribed using the RevertAid H First Strand complementary DNA (cDNA) Synthesis Kit (Thermo Fisher Scientific, Waltham, MA, USA, Lot No. 00648151). The resulting cDNA was used to quantify the expression of COX2, YKL40, CerS1, CerS6, SMPD1, SMPD5 and LAMP1/2.

The cDNA served as a template for amplification in a quantitative PCR (qPCR) reaction, performed using Genaxon GreenMasterMix (2×) (Genaxxon bioscience GmbH, Ulm, Germany, Lot No. M3023.0500) according to the manufacturer’s instructions. Specific primers for the RNA transcripts were designed and synthesized by Integrated DNA Technologies (Leuven, Belgium). Details of the primers are provided in the Supplementary Materials (Table S2, Supplementary File).

Quantitative PCR (qPCR) reactions were conducted using the Rotor-Gene Q 600 system (Qiagen, Germany). The relative expression levels of the target genes were normalized to the mean expression of three housekeeping genes (GAPDH, ACTINβ, hUBC), using the comparative 2^−ΔΔCt^ method. All samples were analyzed in duplicate to ensure accuracy and reproducibility. The qPCR program used is presented in the Supplementary Materials (Table S3, Supplementary File).

### 2.5. TaqMan Analysis

The TaqMan MicroRNA Reverse Transcription Kit (Thermo Fisher Scientific, Waltham, MA, USA, Lot No. 4366596) and TaqMan MicroRNA Assays primers (Thermo Fisher Scientific, Waltham, MA, USA, Lot No. 4427975) were employed to quantify miR-143-3p and miR-181a-5p in WBCs. RNU6 and RNU48 served as endogenous controls.

cDNA specific for miR-143-3p and miR-181a-5p, as well as RNU6 and RNU48, were synthesized using specific 5× stem-loop primers by using 50ng total RNA. Five µL of diluted cDNA (1:7) served as a template in a TaqMan PCR reaction to assess miRNA expression levels. Each reaction contained 5.03 µL of TaqMan Advanced Master Mix (Thermo Fisher Scientific, Waltham, MA, USA, Lot No. 00692208) and 0.47 µL of 20× miRNA primers, with a total reaction volume of 10.5 µL.

PCR reactions were performed on the Rotor-Gene Q 600 machine (Qiagen, Hilden, Germany). The Ct values for each miRNA were normalized to RNU6 and RNU48. All reactions were conducted in duplicate to ensure reliability and precision.

### 2.6. Statistical Analysis

The χ^2^-test or Fisher’s exact probability test was used as methods of contingency table analysis to assess statistical relationships among categorical variables. Analysis of variance or the Mann–Whitney U test was utilized to examine the associations between diagnostic categories and clinical data. We employed a univariate generalized linear model (GLM) to clarify the associations between biomarker data and ASD. The primary analysis is the regression of ADOS on the biomarkers. The findings were appropriately controlled, when necessary, for confounding variables such as age and sex. We used false discovery rate (FDR) p correction to adjust for multiple statistical testing. FDR was performed on the 10 preselected biomarkers. In accordance with the requirements, we implemented various transformations, including logarithmic (log 10) to ensure that our data indicators adhered to a normal distribution. A binary logistic regression analysis (oversampling method) was performed to ascertain the biomarkers that most precisely distinguish between individuals with ASD and control subjects. The odds ratio was computed alongside 95% confidence intervals, in addition to the assessment of accuracy and Nagelkerke metrics, which functioned as the effect size in this analysis. We utilized a systematic combination of automatic and manual methodologies to ascertain the most effective predictors. The automatic method utilized a *p*-value threshold for entry set at 0.05 and a *p*-value threshold for removal established at 0.06. Both manual and stepwise automatic multiple linear regression analyses were employed to examine the influence of biomarkers on clinical rating scales. The automatic, forward stepwise regression method utilized a p-to-enter threshold of *p* = 0.05, whereas a *p*-value of 0.06 was applied for the exclusion of variables from the final regression analysis. The statistical analysis of the model encompassed F statistics along with their associated *p*-values, and the effect size was assessed through R^2^, particularly utilizing the partial Eta squared metric. Furthermore, standardized β coefficients and t-statistics, accompanied by *p*-values, were computed for each variable incorporated in the final regression models. We conducted an analysis of the data to assess the potential presence of multicollinearity and collinearity, utilizing a tolerance limit set at 0.25 and establishing a variance inflation factor threshold of four or greater. The White test, along with a modified iteration of the Breusch–Pagan test, was employed to ascertain the existence of heteroskedasticity. Multivariate normality was assessed by examining the distribution of the regression standardized residuals and using the P–P plot of these residuals. Both types of regression analysis were subjected to a bootstrapping method (1000 bootstraps) to validate the regression models and to check their stability and reliability and to estimate bias by comparing the average bootstrap coefficients with those in the original model. In case there are discrepancies between the bootstrap and the original results, we show the B metrics, bias, SE, significance and 95% confidence intervals obtained via bootstrapping. We also performed automatic linear modeling with the overfit prevention criterion to ascertain that no overfitting is present. All analyses were performed utilizing two-tailed tests, wherein a *p*-value (alpha level) of 0.05 or lower is considered statistically significant. The statistical analysis for this study was performed using version 30 of IBM SPSS Statistics for Windows, Version 25.0 (IBM Corp., Armonk, NY, USA).

## 3. Results

As there are few data on the link between lipid metabolism and ASD, we examined the expression profiles of CerS1, CerS6, SMPD1, SMPD5. We also tested the expression levels of inflammation-related molecules like COX2 and YKL40, along with autophagy-associated markers as LAMP1 and LAMP2. All the above-mentioned mRNAs, together with miR-143-3p and miR-181a-5p, were evaluated in children with ASD compared to typically developing children considered as healthy controls (HC).

The analysis of sociodemographic and clinical data of patients and controls included in the present study showed that ASD patients were somewhat older than controls, but there were no significant differences in the sex ratio (Table 1). Nevertheless, we have adjusted our data for possible effects of sex and age. The intelligence quotient (IQ) was significantly lower in ASD than in control subjects. The total ADOS score was significantly higher in ASD as compared with controls. All scores on the subscales were significantly higher in patients than controls.

When the data from the qPCR for detection of mRNA expression levels were analyzed, they were adjusted for possible effects of age and sex using GLM analysis (age as covariate, sex as second factor). Nevertheless, no significant effects of sex could be detected (even without p correction) and there were no significant group X sex interactions. There were no significant associations between age and any of the variables (even without FDR p correction) either. After FDR p correction, we found increased expression of CerS1, SMPD5, COX2, YKL40, LAMP1, and LAMP2 in ASD as compared with controls. miRNA-181a-5p expression was significantly lower in ASD compared to healthy controls (Table 2).

Table 3 shows the outcome of automatic binary regression analyses performed with ASD as a dependent variable (and heathy controls as a reference group), whilst allowing for the effects of age and sex (Table 3). The automatic step-up method showed that ASD was best predicted by five explanatory variables, namely three of those were positively associated, i.e., COX2, miRNA-143-3p, SMPD5, and two-inversely, i.e., CerS6 and miRNA-181a-5p. These five explanatory variables yielded an accuracy of 92.3% (χ^2^ = 90.71, df = 5, *p* < 0.001), sensitivity = 93.3% and specificity of 90.3% with a Nagelkerke effect size of 0.873. Age and sex were not significant in this regression analysis.

Nevertheless, bootstrapping showed that SMPD5 lost significance, indicating that this biomarker may not have theoretical justification to be included. Therefore, we have re-evaluated the model after removing SMPD5 and show the simplified model in Table 3, step 2 (the bootstrap results).

The four explanatory variables yielded an accuracy of 90.3% (χ^2^ = 72.90, df = 4, *p* < 0.001), sensitivity = 90.0% and specificity of 90.9% with a Nagelkerke effect size of 0.747.

Multiple regression analyses were performed with the rating scale scores as dependent variables and the biomarkers as explanatory variables, whilst allowing for the effects of age and sex (Table 4).

We found that 48.0% of the variance in the total ADOS score was explained by the combined effects of COX2, miRNA-143-3p and CerS1 (all positively associated) and CerS6 and age (inversely associated).

**Figure 2:**
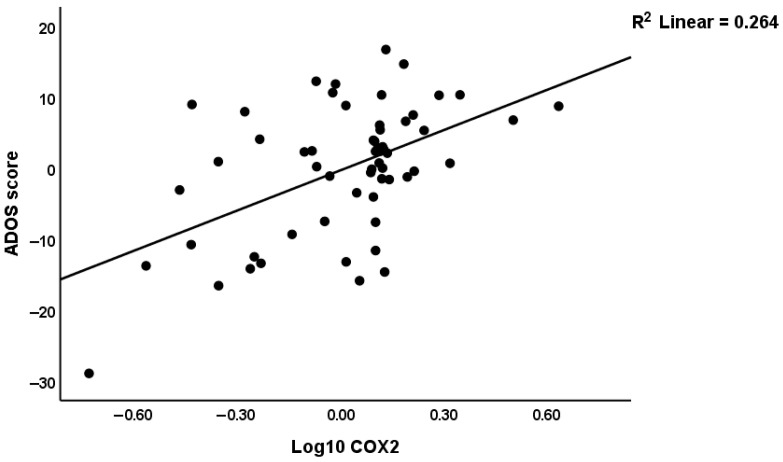
Partial regression of the total ADOS score on COX2 expression.

The partial regression of the total ADOS score on COX2 expression after considering the effects of the other four significant predictors is presented in Figure 2. COX2 expression alone explained 26.4% of the variance in the ADOS score.

**Figure 3:**
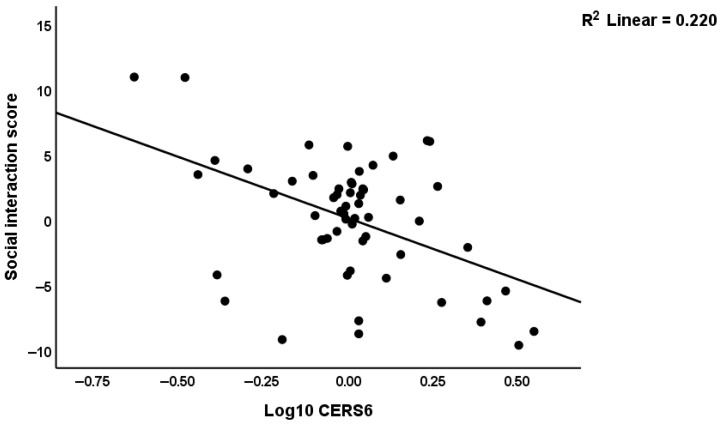
Partial regression of the social interaction score on CerS6.

We found that 28.0% of the variance in communication was correlated with miRNA-181a-5p and age (both negatively associated). The social interaction score was linked to CerS1, COX2, miRNA-143-3p (all positively associated) and to CerS6 and age (inversely associated) which explained 50.5% of the variance. Figure 3 shows the partial regression of the social interaction score on CerS6 after considering the effects of the other explanatory variables.

CerS6 expression was inversely associated with social interaction and explained 22.0% of the variance in the social interaction score. We found that 28.0% of the variance in the play score was explained by the regression on CerS1 (positively associated) and age (inversely correlated) combined. Repetitive behaviors showed an association with increased CerS1 only. All the multiple regression analyses were rerun using 1000 bootstraps.

Comparing the bootstrap coefficients with those in the original models, no major differences were detected. Consequently, the logistic and multiple regression models shown in Table 4 are stable and reliable.

## 4. Discussion

In this study, we investigated the expression profiles of key molecules related to lipid metabolism, inflammation, and autophagy in children with ASD compared to typically developing healthy controls. Our results revealed significantly increased expression of several genes, including CerS1, SMPD5, COX2, YKL40, LAMP1, and LAMP2, alongside a decreased expression of miRNA-181a-5p in ASD patients. Logistic regression modeling identified COX2, miRNA-143-3p, SMPD5, CerS6, and miRNA-181a-5p as the most strongly associated variables with the ASD diagnosis, with the model demonstrating high sensitivity and specificity.

Furthermore, multivariate regression analyses showed that variations in the expression levels of these biomarkers explained a substantial proportion of the variance in clinical features of ASD, including social interaction, communication, and repetitive behaviors. These findings support the hypothesis that disruptions in lipid metabolism, inflammatory processes, and autophagy regulation play a significant role in the pathophysiology of ASD and highlight potential avenues for further research into biomarkers and therapeutic targets.

### 4.1. Novelty

In the present study we report novel data on associations between the expression profiles in WBCs of molecules related to inflammation, autophagy and sphingolipid metabolism with clinical ASD indicators. The current investigation is the first to show results on CerS1 and CerS6 and SMPD5 involved in ceramide generation in ASD. Another interesting outcome of the study is the correlation between clinical assessment scales and LAMP glycoproteins. As the mRNAs of most of the examined molecules are targeted by miRNA-143-3p we focused our attention on this miRNA which has never been examined in ASD.

There are no published data in the literature linking sphingolipid metabolism in blood cells of ASD patients. Wang et al. (2016) carried out a two-phase metabolomics study to pinpoint potential serum biomarkers linked to ASD [43]. Initially, they examined serum samples from a group of 73 children with ASD and 63 healthy individuals, identifying 17 metabolites that showed significant variations. These results were subsequently validated using an independent set of 100 ASD cases and 100 controls, confirming 11 of the 17 metabolites as consistent biomarkers. Among them, sphingosine 1-phosphate and docosahexaenoic acid exhibited a strong correlation with ASD across both cohorts. The findings indicate that certain serum metabolites may serve as dependable biomarkers for diagnosing and assessment of autism [43].

### 4.2. PBMC as a Model in ASD

Sphingolipid metabolism is essential for immune regulation, inflammation, and apoptosis, making PBMCs a valuable model for evaluating changes in these processes. Moreover, PBMCs serve as a convenient, non-invasive model with a phenotype resembling microglia in the CNS. Activated microglial cells are known to release inflammatory and vasoactive molecules, which can compromise the blood–brain barrier and promote immune cell infiltration into the CNS. Research using animal models and postmortem brain tissue from individuals with ASD has revealed microglial activation in multiple brain regions [44,45], along with evidence of BBB dysfunction [46,47].

### 4.3. Ceramide-Generating Enzymes

There are only few very resent studies associating ceramide with autism [6,12,48]. The ceramide-generating enzymes tested in our study, CerS1 and 6 and SMPD1 and 5, have not been shown previously to be implicated in ASD. CerS1 is the main neuronal ceramide synthase isoform [49,50,51]. In addition, it is ubiquitously expressed in the muscle and testis [51]. However, its expression in immune cells is not well studied, underscoring the importance of the current work, which showed that CerS1 messages in immune cells were associated with autism scores (Table 2 and Table 4). Interestingly, CerS6, which has a strong expression in immune cells, showed inverse correlations with parameters related to autism (Table 2 and Table 4; Figure 3) [52]. In a mouse model, CerS6-deficient splenocytes were shown to prevent colitis [53]. The prevention was attributed to the splenocytes ability to proliferate and migrate. Our current data using immune cells show an inverse correlation of CerS6 messages with autism clinical parameters and suggest a possible role of CerS6 in regulating immune response in autism and support further investigation. Deletion of CerS6 has been shown to protect cells from mitochondrial dysfunction caused by oxidative stress and to improve glucose tolerance [54,55,56,57]. This may also help to explain the positive effects of the ketogenic diet in patients with ASD. This type of diet increases the expression of CerS2 and suppresses CerS6 expression [58]. Furthermore, it upregulates genes associated with mitochondrial biogenesis and fatty acid oxidation (e.g., Pgc-1α and Fgf21) and inhibits inflammatory genes (TNF-α, Nf-kb, TLR4, and IL-6) [58].

Currently little is known about the function of SMPD5, also termed as mitochondria-associated sphingomyelinase [29]. Our data show for the first time that SMPD5 messages were increased in immune cells of patients with autism in comparison to the controls (Table 3 and Table 4). Our results suggest that ceramide generation in the mitochondria of immune cells is likely to contribute to autism pathology. Future research can provide information on whether altered ceramide metabolism in immune cells has a direct effect on autism symptomatology. Nevertheless, our current work established that ceramide-generating enzymes are associated with autism and have a potential to serve as biomarkers.

### 4.4. COX2

The results of the present study show increased expression of COX2 in patients with ASD compared to controls. In addition, total COX2 expression alone accounts for 26.4% of the variance in ADOS scores. This strong correlation directly links the inflammatory component of our dataset to clinical manifestation, emphasizing the pathogenic relevance of COX2-mediated signaling in ASD.

Importantly, our findings are consistent with previous reports demonstrating an association between COX2 activity and ASD, including the work of El-Ansary et al. (2024), who identified COX2 and its downstream metabolites as part of a biomarker panel indicating ASD severity [59].

This is not surprising, as COX enzymes are key for the production of prostaglandins, which are important regulators of inflammation and neuronal activity in the brain and could explain the changes observed in ASD. Moreover, arachidonic acid, the primary substrate for COX2, has been reported to be altered in individuals with ASD, suggesting that dysregulated substrate availability may further amplify COX2-mediated inflammatory signaling [60]. In addition, the altered expression of COX2 leads to oxidative stress, deregulation of mitochondrial functions, and imbalance of neurotransmitters and intestinal flora [61,62], which are prevalent in the pathogenesis of autism.

Our findings, in agreement with previous reports, suggest that COX2 overexpression in peripheral immune cells reflects a systemic pro-inflammatory in ASD. This may contribute to both peripheral immune dysregulation and central neuroinflammation.

Normal COX2/PGE2-mediated signaling regulates dendritic spine formation, synaptic plasticity, and memory [63,64]. Therefore, alterations in COX2 levels have been linked to various neurological and neurodevelopmental diseases, including ASD [62].

Several studies have reported an association of clinical indicators in ASD with expression levels [65,66], or with gene polymorphisms. A similar finding is reported in a genetic study in Korean children. The results showed significant differences in overactivity/agitation by the ADOS scale and in communication by the ADI-R depending on the genotype. The A allele of rs2745557 was found to prevail in individuals with ASD [67]. The consistency between our regression model and previous genotype–phenotype correlations further supports the contribution of COX2 to behavioral variability in ASD. According to a study of El-Ansary et al. autistic patients with different degrees of sensory impairment can be stratified based on a set of six markers, among which are PGE2 and COX2. The strong correlation established between COX2 expression, and sensory scores implies that COX2 metabolites may modulate the function of sensory nerves and pain response [68], leading to behavioral changes characteristic for ASD [69]. Our data confirms that COX2 correlates with ADOS subscales, strengthening the evidence that COX2 metabolites modulate sensory and behavioral responses in ASD. Prostaglandin E2 (PGE2) is known to stimulate glutamate release from glial cells and to modulate the activity of neighboring neurons [70]. This may help explain the behavioral abnormalities in autistic children, including our target cohort. COX2, PGE2 and four other lipid mediators are suggested as a viable cluster of biomarkers for predicting high sensory abnormalities seen in autistic patients [69]. Qasem (2018) found elevated COX2 and PGE2 in autistic patients, which were negatively correlated with the SSP score [71]. All these examples are supported by our data of multiple regression analyses with the ADOS scale scores, as well as by the established positive association of COX2 with ADOS row scores in ASD.

In addition to immune and neuronal effects, accumulating evidence indicates that COX2-mediated inflammatory signaling may also interact with gut function and intestinal microbiota composition, further contributing to systemic inflammation and behavioral manifestations in ASD [72,73].

The involvement of COX2 in the pathogenesis of neuropsychiatric disorders is further reinforced by the positive effect of COX2 inhibitors observed in patients with schizophrenia and major depressive disorder [74]. COX2 inhibitors are suggested to influence positively the neurodevelopment in ASD by modulating the Wnt-signaling pathway and the dendritic arborization [66]. However, this relationship is not yet fully understood and further studies are needed to reveal whether COX2 inhibition could have a therapeutic effect in ASD.

### 4.5. YKL40

Although YKL40’s functions are not completely clarified yet, it may negatively modulate neurotrophic factor-related changes in neuronal repair and regeneration [75]. We determined an increased expression of YKL40 in ASD as compared to controls. Several studies revealed a positive correlation between YKL40 levels and the severity of diseases, including ASD, where neuroinflammation plays a central role in their pathophysiology [76,77]. Furthermore, Demirci et al. (2024) reported that YKL40 could significantly predict ASD severity, indicating a potential role of neuroinflammation in the development of ASD even though they found no evident differences in mean serum YKL40 levels between patients and controls [76]. Our results are also consistent with the hypothesis that YKL40 plays an important role in neuronal degeneration and is related to local neuroinflammation as supported by our data on COX2 expression.

### 4.6. LAMPS

In this study, we identified a two-fold increase in LAMP1 expression and a three-fold increase in LAMP2 expression in ASD patients compared to HC. Our findings extend those of Deng et al. (2023), who reported intense LAMP1 expression in cerebellar samples from ASD patients [78]. They highlighted the potential impact of LAMP1 on neurodevelopment and its role in regulating immune cells in individuals with autism [78].

Moreover, immune dysregulation is one of the most observed abnormalities in ASD, characterized by alterations in T cells, B cells, and NK cells, along with increased levels of inflammatory cytokines, autoantibodies, and microglial activation [78]. These immune disturbances may be linked to abnormal LAMP1 expression, which affects NK/T cell activity and contributes to immune imbalance [78]. Interestingly, in Alzheimer’s disease LAMP1 expression is upregulated in neurons with granulovacuolar degeneration, microglia, and multinucleated giant cells [79].

Similarly, elevated LAMP2 expression has been reported in lysosomal storage diseases and under conditions of high sucrose levels, which are associated with the accumulation of undigested cellular materials [80,81]. Increased lysosomal protein expression has also been linked to abnormal lipid metabolism caused by lysosomal accumulation [80,82].

Intriguingly, LAMP1 and LAMP2 may have additional critical functions in mammals, because their deletion in mutant mice leads to embryonic lethality [83]. One example of this is that the abundance of lysosomal proteins increases as a result of oxidative stress. LAMP2 contributing to the ROS clearance [84,85]. Zhang et al. (2023) found that oxidative stress induces lysosomal membrane permeabilization and ceramide accumulation [85]. In response to lysosomal damage, cells activate compensatory mechanisms by regulating lysosomal biogenesis via the mTORC1-TFEB pathway [86]. Taken together, these findings highlight the crucial role of lysosomal proteins in ASD pathophysiology. Furthermore, the involvement of LAMP1 and LAMP2 in autophagy suggests that their disruption may lead to cellular dysfunction, accumulation of defective organelles, and impaired waste clearance.

### 4.7. miR-181a-5p and miR-143-3p

We focused our research on two selected miRNAs, miR-181a-5p and miR-143-3p. They are involved in inflammation, mitochondrial dysfunction, and metabolism [87,88]. While miR-181a-5p expression shows controversial expression across different autistic tissues, no data are currently available for miR-143-3p [34].

Our results revealed a significantly reduced expression of miR-181a-5p in the autistic group compared to the control group.

Increased miR-143-3p expression was in the logistic regression analysis significantly and positively associated with ASD, and miR-143-3p expression was positively associated with the behavioral changes in ASD.

It has been established that miR-181a-5p is highly expressed in the brain and plays a crucial role in neurodevelopment and neurodegeneration [86]. Increased expression in neural tissue has been linked to cerebral ischemic injury [38,89], whereas reduced expression appears to have a protective effect [90]. Additionally, miR-181a-5p regulates mitochondrial activity [87,91,92], synaptic plasticity [93] and contributes to cell differentiation [94], TNFα modulation and NK cell differentiation [95]. It is also a key regulator of autophagy, which may explain its connection to LAMP proteins [96]. Key targets of miR-181a-5p, are an essential component of the synaptosomal-associated protein 25 (SNAP-25), which facilitates synaptic vesicle fusion and neurotransmitter release [97]. By modulating SNAP-25 expression, miR-181a-5p influences synaptic plasticity and overall neuronal signaling. Another gene under the regulation of miR-181a-5p, the C-X-C motif chemokine ligand 12 (CXCL12), is critical for neuronal migration and synaptic transmission [98]. Through its interaction with CXCL12, miR-181a-5p contributes to neuronal injury response mechanisms and may have broader implications for synaptic function. Additionally, miR-181a-5p serves as a key modulator of immune responses and inflammation by targeting cytokine-related genes, including tumor necrosis factor-alpha (TNF-α), interferon-gamma (IFN-γ), and sirtuin-1 (SIRT1). This regulation helps maintain the equilibrium between pro-inflammatory and anti-inflammatory signaling [99,100]. When miR-181a-5p expression is reduced, excessive immune activation and persistent inflammation in the brain may occur, potentially leading to synaptic dysfunction, disrupted neuronal connectivity, and cognitive impairments, all of which are characteristic of ASD. Moreover, miR-181a-5p plays a significant role in microglial activity regulation, as microglia functions as immune cells responsible for maintaining brain homeostasis [101]. Research conducted by Agostini et al. demonstrated that miR-181a-5p also influences key neurotransmitter systems, including dopaminergic and glutamatergic pathways, both of which are vital for cognitive processes and behavior [97]. Altered miR-181a-5p expression may disrupt the balance between excitatory and inhibitory neurotransmission, contributing to ASD-related symptoms. Furthermore, given its essential role in hippocampal function, diminished levels of miR-181a-5p may impair learning and memory. Studies suggest that this microRNA is closely linked to neural plasticity, which is fundamental for cognitive development and memory retention [97].

The effects of miR-181a-5p are highly context-dependent, as excessive expression has been linked to acute neuronal injury, whereas moderate downregulation may be neuroprotective. It was shown that sustained reduction can promote persistent neuroinflammation and impair synaptic plasticity, learning, and memory [89,90,91,92,93].

Our results show a significant association between miR-143-3p and the ADOS scale in ASD. miR-143-3p strongly inhibits COX2 expression by directly binding to its 3′-UTR, thereby influencing inflammatory processes [102]. Interestingly, miR-143-3p also targets pyruvate carboxylase (PC) by binding to the 3′-UTR of human PC mRNA, further implying its role in metabolic regulation [103]. This miRNA is brain-specific and regulates autophagy during embryonic development [104,105] but has not been studied in ASD.

The association between miR-143-3p expression and ADOS scores suggests its potential as a biomarker for autism severity. However, it is crucial to highlight that no published data currently link miR-143-3p to ASD in the literature. Therefore, it could provide fresh perspectives on the molecular mechanisms underlying ASD and serve as a promising biomarker for assessing symptom severity. Further research is necessary to determine whether miR-143-3p plays a direct role in ASD pathology or if its altered expression results from broader neurodevelopmental abnormalities.

### 4.8. Multiple Molecules Signatures Associated with the ADOS Scale Scores as Dependent Variables in ASD

Early screening and diagnosis of ASD are crucial for initiating therapy and improving prognosis. Multiple regression analyses with ADOS scale scores as dependent variables revealed that 48.0% of the variance in the total ADOS score is explained by the combined effects of COX2, miRNA-143-3p, and CerS1 (all positively associated) and CerS6 and age (inversely associated).

This integrated model highlights a convergent molecular axis linking lipid metabolism (CerS1/CerS6), inflammation (COX2), and post-transcriptional regulation (miR-143-3p). The directionality of these associations implies that excessive ceramide production and inflammatory activation exacerbate ASD symptoms, whereas CerS6 activity and age-related compensations may counterbalance these effects. The results are further supported by evidence for CerS1 inactivating mutations leading to cerebellar ataxia in humans and mice, and epilepsy with intellectual disability in humans (a rare disease; progressive myoclonic epilepsy type 8) [106,107] (https://www.orpha.net/en/disease/gene/CERS1; accessed on 3 September 2024) CerS1 was also identified as one of the three genes necessary for healthy aging [106]. Even more compelling are the data on CerS6 from an animal model of multiple sclerosis, where it has been identified as a key determinant of disease severity [54]. A neuroprotective role is suggested for CerS6, likely due to neuron-specific deletion of C16-synthesizing enzymes, which protect against mitochondrial dysfunction caused by oxidative stress [54]

Several studies have also reported a link between autistic behavior and COX2 levels, highlighting the involvement of disrupted COX2/PGE2 signaling in ASD pathophysiology [63,108,109]. The study of Ramer et al. (2003) demonstrated that R(+)-methanandamide enhances COX2 expression in human neuroglioma cells by raising ceramide levels, which in turn activates mitogen-activated protein kinase (MAPK) signaling [110]. Blocking ceramide synthesis prevents this effect, emphasizing the crucial role of ceramide in regulating COX2 [110]. In this regard, it is worth mentioning that ceramide synthesis upregulates COX2, suggesting that some of the tested biomarkers are co-regulated.

This evidence reveals abnormalities in lipid mediators and metabolism associated with the pathogenesis of ASD [111]. More importantly, lipid metabolism-related abnormalities are amenable to therapeutic intervention [111].

## 5. Limitations

The main limitation of our study is the small number of healthy controls. The disparities across groups are not the principal outcome; the key statistical method employed in this study is the regression of ADOS on the biomarkers. The minimum a priori sample size was calculated using G*Power 3.1.9.7. Utilizing an effect size of 0.25 (about 20% explained variance), with an alpha of 0.05, power of 0.8, and a maximum of 5 covariates, the minimum sample size required is 58. The actual post hoc power calculated for this regression is 0.99.

Other limitations are the use of PBMCs as a non-invasive and easily accessible cell source in ASD patients, and the lack of protein-level validation of the observed molecular changes.

## 6. Conclusions

In conclusion, the present study reports for the first time data on altered expression profiles of molecules related to lipid metabolism and lysosomal membrane proteins in patients with ASD. We observed increased mRNA levels of CerS1, SMPD5, COX2, YKL40, LAMP1, and LAMP2 and decreased expression of miRNA-181a-5p in ASD patients compared to HC. Additionally, we identified a correlation between CerS1, CerS6, COX2, and miRNA-143-5p with ADOS scores. Multiple regression analysis revealed that 48.0% of the variance in the total ADOS score was explained by the combined effects of COX2, miRNA-143-3p, and CerS1 (all positively associated) and CerS6 and age (inversely associated). These results provide new insights into molecular alterations associated with ASD and may reinforce future studies aimed at clarifying their functional relevance.

## Figures and Tables

**Table 1:** Socio-demographic and clinical data of ASD patients and health controls (HC).

Variables	HC n = 10	ASD n = 60	F/x^2^/U	Df	*p*
Age	5.8 (2.4)	4.0 (2.2)	F = 5.80	1/68	0.019
IQ *	98.4 (4.3)	55.2 (1.8)	F = 79.58	1/57	<0.001
Sex (n, M/F)	6/4	50/10	x^2^ = 2.91	1	0.088
ADOS	0.64 (0.68)	24.9 (0.70)	U	-	<0.001
Communication	0.33 (0.50)	5.70 (0.22)	U	-	<0.001
Social interaction	0.29 (1.02)	13.76 (0.44)	U	-	<0.001
Play	0.07 (0.40)	4.82 (0.17)	U	-	<0.001
Repetitive behavior	0.04 (0.56)	5.76 (0.24)	U	-	<0.001

Legend: Results are shown as mean (SD). F: results of analysis of variance; x^2^: results of contingency analysis; U = Mann–Whitney U test shown age and sex adjusted values. * Adjusted for sex and age. Abbreviations: n, M/F-number of males/females.

**Table 2:** Biomarker data in ASD patients and healthy controls (HC).

Variables	HC n = 10	ASD n = 60	F	Df	*p*	FDR *p* Value
CerS1 *	1.36 (3.96)	9.35 (1.98)	7.10	1/65	0.010	0.020
CerS6 *	1.32 (0.82)	0.82 (0.07)	2.27	1/65	0.136	0.170
SMPD1	1.18 (0.16)	1.20 (0.08)	0.27	1/65	0.274	0.304
SMPD5 *	1.14 (1.28)	4.30 (0.46)	7.47	1/65	0.008	0.020
COX2 *	1.17 (0.55)	2.93 (0.28)	10.81	1/65	0.002	0.010
YKL40	1.01 (2.15)	2.15 (1.69)	5.25	1/65	0.025	0.036
LAMP1	1.24 (0.46)	2.50 (0.23)	5.88	1/65	0.018	0.030
LAMP2	1.61 (0.99)	4.53 (0.36)	7.32	1/65	0.009	0.020
miRNA-143-3p *	1.53 (0.30)	1.10 (0.15)	0.77	1/65	0.383	0.383
miRNA-181a-5p *	6.48 (0.94)	0.92 (0.47)	17.40	1/65	<0.001	0.010

Legend: All results of univariate GLM analysis adjusted for age with sex as a second factor. There were no significant differences between boys and girls in any of the biomarkers. * Processed in Log 10 transformation. Values are presented as fold change in gene expression.

**Table 3:** Results of logistic binary regression analysis with ASD as dependent variable.

	Biomarkers		B	SE	Wald		*p*	OR	Lower	Upper
Step 1	CerS6		−3.021	0.857	12.434		<0.001	0.049	0.009	0.261
	COX2		3.201	1.116	8.223		0.004	24.563	2.754	219.041
	miRNA-143-3p		2.296	0.867	7.017		0.008	9.933	1.817	54.297
	miRNA-181-5p		−2.322	0.947	6.017		0.014	0.098	0.015	0.627
	SMPD5		2.513	0.970	6.709		0.010	12.345	1.843	82.687
			B	Bias		Std. Error		Sig. (2-tailed)	95% Confidence Interval	
									Lower	Upper
Step 2		CERS6	−1.905	−6.493		56.366		<0.0001	−11.793	−1.290
		COX2	2.361	7.320		67.003		<0.001	1.704	8.583
		miRNA-143	1.665	4.300		37.877		<0.001	1.118	6.624
		miRNA-181	−2.070	−7.929		71.336		0.014	−8.885	−0.667

Step 1: original logistic regression analysis. Step 2: bootstrapped logistic regression analysis; the results are based on 1000 bootstrap samples. Legend: OR: Odds ratio.

**Table 4:** Results of multiple regression analyses with the ADOS scale scores as dependent variables.

Dependent Variables	Experimental Variables	β	t	*p*	R^2^	F	df	*p*
ADOS	Model	-	-	-	0.480	9.79	5/53	<0.001
	COX2	0.550	4.38	<0.001				
	Age	−0.315	−3.17	0.003				
	CerS6	−0.350	3.29	0.002				
	miRNA-143-5p	0.399	3.27	0.002				
	CerS1	0.303	2.62	0.011				
Communication	Model	-	-	-	0.280	10.67	2/55	<0.001
	Age	−0.433	−3.78	<0.001				
	miRNA-181-5p	−0.318	−2.77	0.007				
Social interaction	Model	-	-	-	0.505	10.59	5/52	<0.001
	CerS1	0.353	3.01	0.004				
	Age	−0.315	−3.19	0.002				
	CerS6	−0.396	−3.82	<0.001				
	COX2	0.425	3.36	0.001				
	miRNA-143-5p	0.308	2.54	0.014				
Play	Model	-	-	-	0.280	10.71	2/55	<0.001
	CerS1	0.380	3.32	0.002				
	Age	−0.353	−3.08	0.003				
Repetitive behavior	Model	-	-	-	0.157	10.46	1/56	0.002
	CerS1	0.397	3.23	0.002				

## Data Availability

The original contributions presented in this study are included in the article/Supplementary Materials. Further inquiries can be directed to the corresponding authors.

## Supplementary Materials

The following supporting information can be downloaded at: https://www.mdpi.com/article/10.3390/biom16020303/s1, Table S1: Characteristics of CerS (1–6); Table S2: Primer sequences used for qPCR analysis; Table S3: qPCR Program.

## Abbreviations

3′-UTR	3′ Untranslated Region
ADOS	Autism Diagnostic Observation Schedule
ASD	Autism spectrum disorder
ASM	Acid Sphingomyelinase
cDNA	Complementary DNA
CerS1	Ceramide synthases 1
CerS6	Ceramide synthases 6
CNS	Central Nervous System
COX2	Cyclooxygenase 2
DSM-5	Diagnostic and Statistical Manual of Mental Disorders
IQ	Intelligence Quotient
KD	Ketogenic diet
LAMP1	Lysosome-associated membrane proteins 1
LAMP2	Lysosome-associated membrane proteins 2
MAPK	Mitogen-Activated Protein Kinase
miR-143-3p	MicroRNA-143
miR-181a-5p	MicroRNA-181
mTOR	Mechanistic Target Of Rapamycin
PGE2	Prostaglandin E2
qPCR	Quantitative PCR
ROS	Reactive Oxygen Species
SMPD1	Lysosomal acid sphingomyelinase
SMPD5	Sphingomyelin phosphodiesterases 2
WBCs	White Blood Cells
YKL40	Chitinase-3-like protein 1
