# Supplementary material for: Association of Clinical Severity in Autism Spectrum Disorder with Biomolecules Involved in Lipid Metabolism, Inflammation and miRNAs
Source: Biomolecules. 2026 Feb 14;16(2):303. doi: 10.3390/biom16020303 (PMC12938623; doi:10.3390/biom16020303)
Supplement: Supplementary file 1 [file biomolecules-16-00303-s001.zip › biomolecules-4137676-supplementary.pdf]

Table S1. Characteristics of CerS (1–6)

Isoforms	Chromosomal location	Gene size (base pairs)	Acyl chain-length specificity	Location of the CerS protein
CerS1	19p12	25.837	C18	Endoplasmic reticulum (ER)
CerS2	1q21.2	9.792	C20-C26	ER
CerS3	15q26.3	144.326	C22-C26	ER
CerS4	19p13.2	53.046	C18-C20	ER
CerS5	12q13.3	37.565	C16	ER
CerS6	2q24.3	318.394	C14 and C16	Mitochondria

Table S2. Primer sequences used for qPCR analysis

Gene	Primer 5’-3’	Length	Tm	GC%
SMPD1	Forward CTGAGGATCGAGGAGACAAAG	21	54.5	52.4
	Reverse ACCTGGCTACAATTCGGTAATA	22	53.5	40.9
SMPD5	Forward TGCAAGCACTGGCTGAA	17	55	52.9
	Reverse CGACCTCTCCACTTTGTTCTC	21	62	52.4
CerS1	Forward ACGCTACGCTATACATGGACAC	22	56.7	50
	Reverse AGGAGGAGACGATGAGGATGAG	22	57.5	54.5
CerS6	Forward GGGATCTTAGCCTGGTTCTGG	21	57.2	57.1
	Reverse GCCTCCTCCGTGTTCTTCAG	20	57.8	60
COX2	Forward TCCTAGTCCTCATCGCCCTC	20	57.9	60
	Reverse AGATTAGTCCGCCGTAGTCG	20	56.2	55
YKL40	Forward CTGCTCCAGTGCTGCTCT	21	60.3	61.9%
	Reverse TACAGAGGAAGCGGTCCAAGG	25	59.8	48%
LAMP1	Forward CTCTAATGTCTGCAGCTCAAGG	22	55.4	50%
	Reverse TGTACACAGCGCAGAACAGG	20	57.6	55%
LAMP2	Forward ACAACAGTGGATCAGACAGTACG	23	56.5	47.8%
	Reverse	21	55.8	47.6%

	AGCAGCAAGCATCAGTTCTTC			
GAPDH	Forward AGGTGAAGGTCGGAGTCAACG	21	59	57.1
	Reverse GCTCCTGGAAGATGGTGATGG	21	57.7	57.1
ACTINβ	Forward AGTGTGACGTGGACATCCGGA	21	59.3	54.5
	Reverse GCCAGGGCAGTGATCTCCTCCT	22	60.3	57.9
hUBC	Forward TCCTGATCAGGCAGAGGTTGATCTT	25	59.5	48
	Reverse GGACCAAGTGCAGAGTGGACTCTT	24	60.7	54.1

Table S3. qPCR Program

Cycle Step	Temperature	Time	Cycles
Initial Denaturation	95 °C	60 sec.	1
Denaturation<br>Extension	95 °C,<br>60 °C	15 sec.<br>30 sec.	40-45
Melt Curve	60–95°C		1
